# An international collaborative study to co-produce a patient-reported outcome measure of cardiac arrest survivorship and health-related quality of life (CASHQoL): A protocol for developing the long-form measure

**DOI:** 10.1016/j.resplu.2022.100288

**Published:** 2022-08-31

**Authors:** Kirstie L. Haywood, Charlotte Southern, Elizabeth Tutton, Paul Swindell, David Ellard, Nathan A. Pearson, Helen Parsons, Keith Couper, Katie N. Daintyi, Sachin Agarwal, Gavin D. Perkins, SURViVORS PROM Buddies Group, Kristofer Arestedt, Theresa Aves, Janet Bray, Anne Brookes, Clifton Callaway, Maaret Castren, Marcus Eng Hock Ong, Katrysha Gellis, Paulien H. Goossens, Jan-Thorsten Graesner, Angela Hartley, Rob Hoadley, Johan Israelsson, David Jeffrey, Vicky Joshi, Thomas R. Keeble, Gisela Lilja, John Long, Marco Mion, Laurie J. Morrison, Veronique R.M. Moulaert, Diane Playford, Kelly Sawyer, Federico Semeraro, Karen Smith, Barry Williams, Jasmine Wylie

**Affiliations:** a Warwick Research in Nursing, Warwick Medical School, University of Warwick, Gibbet Hill, Coventry CV4 7AL, United Kingdom; b Doctoral Student. Warwick Research in Nursing, Warwick Medical School, University of Warwick, Gibbet Hill, Coventry CV4 7AL, United Kingdom; c Kadoorie, Oxford Trauma and Emergency Care, Nuffield Department of Orthopaedics, Rheumatology and Musculoskeletal Sciences, University of Oxford, and Oxford University Hospitals NHS Foundation Trust, United Kingdom; d Founder and Chair Sudden Cardiac Arrest UK (SCA-UK), United Kingdom; e Warwick Clinical Trials Unit, Warwick Medical School, University of Warwick, Gibbet Hill, Coventry CV4 7AL, United Kingdom; f University Hospitals Coventry and Warwickshire, Clifford Bridge Road, Coventry CV2 2DX, UK; g Warwick Research in Nursing, Warwick Medical School, University of Warwick, Gibbet Hill, Coventry CV4 7AL, United Kingdom; h Warwick Clinical Trials Unit, Warwick Medical School, University of Warwick, Gibbet Hill, Coventry CV4 7AL, United Kingdom; i Warwick Clinical Trials Unit, Warwick Medical School, University of Warwick, Gibbet Hill, Coventry CV4 7AL, United Kingdom; j Critical Care Unit, University Hospitals Birmingham NHS Foundation Trust, Birmingham B9 5SS, United Kingdom; k North York General Hospital, Toronto, Canada; l Institute of Health Policy, Management and Evaluation, University of Toronto, Toronto, Canada; m Department of Neurology, Division of Critical Care & Hospitalist Neurology, Columbia University Irving Medical Center, New York Presbyterian Hospital, New York 10032, United States; n Warwick Clinical Trials Unit, Warwick Medical School, University of Warwick, Gibbet Hill, Coventry CV4 7AL, United Kingdom; o Critical Care Unit, University Hospitals, Birmingham B9 5SS, United Kingdom

**Keywords:** Cardiac arrest recovery, Survivorship, Health-related quality of life, Measurement, Outcome assessment, Patient-reported outcomes, Co-production

## Abstract

**Background:**

Current measures of health-related quality of life are neither sufficiently sensitive or specific to capture the complex and heterogenous nature of the recovery and survivorship associated with cardiac arrest. To address this critical practice gap, we plan a mixed-methods study to co-produce and evaluate a new cardiac arrest-specific patient/survivor-reported outcome measure (PROM).

**Methods:**

International guidelines have informed a two-stage, iterative, and interactive process.

Stage one will establish what is important to measure following cardiac arrest. A meta-ethnography of published qualitative research and a qualitative exploration of the experiences of survivors and their key supporters will inform the development of a measurement framework. This will be supplemented by existing, extensive reviews describing concepts that have previously been measured in this population. Focus groups with survivors, key supporters, and healthcare professionals, followed by further interviews with survivors and key supporters, will inform the iterative refinement of the framework, candidate items, and PROM structure.

Stage two will involve a psychometric evaluation following completion by a large cohort of survivors. Measurement theory will inform: the identification of items that best measure important outcomes; item reduction; and provide robust evidence of measurement and practical properties.

**Discussion:**

An international, collaborative approach to PROM development will engage survivors, key supporters, researchers, and health professionals from study commencement. Successful co-production of the cardiac arrest survivorship and health-related quality of life (CASHQoL) measure will provide a robust, relevant, and internationally applicable measure, suitable for completion by adult survivors, and integration into research, registries, and routine care settings.

Ethical approval: University of Warwick Biomedical & Scientific Research Ethics Committee (BSREC 22/20-21 granted 10/11/20).

## Introduction

Advances in resuscitation science have targeted different stages in the chain of survival including event recognition, community programmes and post-resuscitation care, contributing to steady growth in the number of people surviving cardiac arrest.^1^ There are large regional variations in survival rates to both hospital discharge (global average 8.8%, 95% confidence interval 8.2–9.4%) and one-year survival (global average 7.7, 95% confidence interval 5.8–9.5%).^2^ In the USA, this equates to approximately 36,500 survivors a year.^3^ However, even when the heart can be restarted, the neurological and cardiac damage caused by both the original event and subsequent treatment can have substantial long-term psychological, cognitive, physical, and social consequences.^1^, ^4^, ^5^, ^6^

Historically, outcome reporting following cardiac arrest has typically utilised crude clinician-reports of functional survival, such as the Cerebral Performance Category Scale (CPC).^7^ However, such broad-based assessments are not designed to consider how survivors experience the impact of the cardiac arrest.^1^, ^4^, ^5^, ^6^ Recent guidance highlights the importance of assessing the survivor’s health-related quality-of-life (HRQoL).^8^ Well-developed patient-reported outcome measures (PROMs) are single or multi-item questionnaires, that ‘extend patient outcome beyond survival’,^9^ seeking to assess health outcomes that are most important to the patient – i.e., how they feel, function, and live their lives.^10^ The relevance or content validity of a PROM is, therefore, of utmost importance,^9^ enhancing their utility for routine practice, audit and research settings.

In the absence of a cardiac arrest survivor-specific PROM,^11^ generic measures have been recommended.^8^ However, such measures lack specificity to the experience of cardiac arrest survivors, and may underestimate the substantial burden associated with survival.^4^, ^5^, ^8^ Assessment guidance, therefore, recommends their use alongside measures that are condition or concept ‘specific’ (e.g., fatigue).^12^ Thus, there is an increased relevance of an enhanced and well-developed condition-specific measure which would improve patient acceptability, clinical utility, and responsiveness to important changes in health.

This protocol describes the methodology for the collaborative co-production of the first cardiac arrest survivors PROM – the Cardiac Arrest Survivorship and Health-related Quality of Life (CASHQoL) questionnaire.

## Methods

We aim to co-produce a patient-reported measure of cardiac arrest survivorship and HRQoL for adult survivors of in-hospital or out-of-hospital cardiac arrest, that is relevant, robust, and internationally applicable in clinical research, routine practice, and epiregistries.

Development will be underpinned by measurement theory and international guidance.^9^, ^10^, ^13^, ^14^, ^15^, ^16^, ^17^, ^18^ It will be driven by an iterative process of shared learning, co-production and active collaboration with an international group of survivors, key supporters (i.e., a family member/friend impacted by the cardiac arrest and/or subsequent recovery) and advocates, researchers, and health professionals with expertise in post-cardiac arrest care and/or research (Appendix 1)^10^, ^17^:

• *Core team:* six methodologists (UK: KH, CS, ET, NP, HP; Canada: KD) and three health professionals (UK: GP, KC; USA: SA)
• *Public Partners (PP):* four cardiac arrest survivors (PS, DJ, DE, AB), one key supporter (BW), and one advocate (JL) will ensure that the survivor’s voice is considered throughout the development process.
• *International Advisory Group (IAG):* representatives from 11 countries (Australia, Canada, Denmark, Finland, Germany, Italy, the Netherlands, Singapore, Sweden, United Kingdom, USA), including three additional survivors and key supporters, health professionals (17) and researchers (3) will provide cross-cultural input and project oversight. They will contribute to recruitment, data collfection and analysis.

**Fig. 1:**
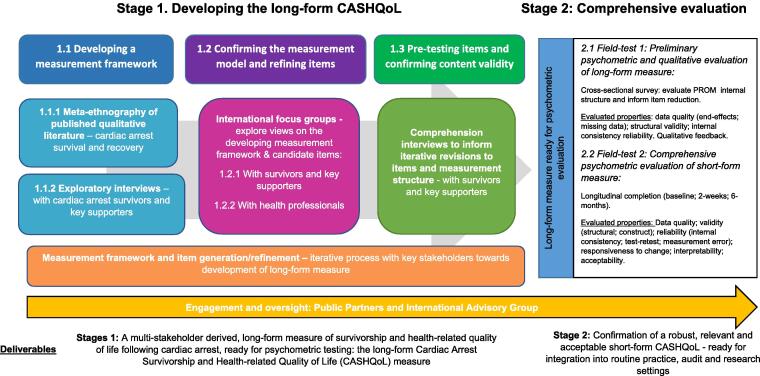
Flow diagram showing the stages in the co-production of the CASHQoL.

PROM development will involve a two-stage, non-linear, mixed-methods approach, whereby both qualitative and quantitative activities are iteratively and interactively utilised (Fig. 1).^9^, ^10^, ^19^ This protocol will detail stage one - developing the long-form CASHQoL. Stage two – a comprehensive psychometric and qualitative evaluation – will be reported at a later date*.*

## Stage 1: Developing the long-form CASHQoL

Three development activities:^9^, ^10^, ^13^, ^16^, ^19^.

***Developing a measurement framework:*** clarifying what to measure.
***Confirming the measurement framework and refining items:*** refining PROM focus and content.
***Pre-testing items and confirming content validity:*** ensuring relevance, acceptability, comprehensiveness and comprehensibility.

### Developing a measurement framework

An essential first step in PROM development is the measurement framework which describes a hypothesised structure for the new measure, the concept(s) of health to be assessed, and potential associations between patient important outcomes (‘domains’).^9^, ^10^, ^19^ The framework is continually developed and refined as data is collected.

Reference to recent extensive reviews of recovery and survivorship^1^, ^4^, ^6^ international guidelines,^5^, ^20^ and outcome reporting^8^, ^11^ will inform a developing list of patient important outcomes and potential domains, and a preliminary measurement framework.

Qualitative research is an essential component of this stage,^21^, ^22^ supporting exploration of the outcomes that really matter to survivors, their key supporters, and healthcare professionals, and informing refinement of the measurement framework. We will, therefore, systematically review relevant, international qualitative literature and conduct interviews with survivors and their key supporters.

#### A meta-ethnography of published qualitative research^23^, ^24^

Noblit and Hare’s seven stages to meta-ethnography will be adopted.^24^

Systematic electronic searches of four major databases (Medline (OVID), EMBASE (OVID), PsycINFO, and CINAHL) will be undertaken to identify published qualitative studies of cardiac arrest survival which provide the views of survivors and their key supporters. An established search strategy for meta-ethnographic reviews^25^ will be combined with medical subject headings (MeSH), keywords, and free-text terms relevant to cardiac arrest, recovery and survivorship. One reviewer will screen all titles and abstracts against inclusion criteria (CS); a 10% subset will be second assessed (NP). A further 10% of all full-text titles will also be second assessed (NP).

Included papers will be independently read by two reviewers (CS, KH) who will: (i) critically appraise the quality of each paper^26^ and (ii) extract all second-order concepts – i.e., the author’s interpretation of the qualitative data. A collaborative interpretation of each concept will then be established (CS, KH). Confidence in the findings of the review will be assessed.^27^

A six-person review team (one PP (DE), one health professional (KC), and four researchers (ET,NP,KS,KD)) will independently review all collaborative interpretations and iteratively abstract them into categories (or ‘piles’ of related concepts). The group will discuss and confirm the categories, and identify overarching themes. A conceptual model of how the themes relate to each other and a line of argument will be established, contributing to further refinement of the measurement framework.

#### Exploratory interviews

Exploratory interviews with cardiac arrest survivors and their key supporters will be conducted to understand their experiences of survival, recovery, and survivorship. Semi-structured interviews will be conducted to support an exploration of participants’ experiences of cardiac arrest and the recovery journey.^21^, ^22^, ^28^

#### Participants, setting, and recruitment

Adults (18 years and above) will be eligible for inclusion if they, or a spouse/partner/key supporter, have experienced a cardiac arrest in the previous three to 36-months, and can share their experiences about recovery (Table 1). Where survivors and their key supporters agree to participate, they may be interviewed together or separately.^28^ A convenience sample will be recruited, striving for maximum variation across a range of socio-demographic variables (age, gender, time since arrest, cognitive function, marital status, geographical location, socioeconomic status, and ethnicity). Participants will provide verbal informed consent at the time of the interview.

**Table 1:** Eligibility criteria and recruitment processes for qualitative activities in Stage 1 of PROM development.

Eligibility Criteria		Recruitment Process	
Participants	Inclusion criteria	Country participation	Sampling
Stages 1.1.2 (interviews), 1.2.1 (focus groups), and 1.3 (three-step interviews)			
Adult (18 years and above) cardiac arrest survivors; cardiac arrest in previous three to 36-months		Stage 1.1 (interviews): participants from four countries (UK, USA, Canada, Australia)	Convenience sample
Adult (18 years and above) key supporter^a^ of a cardiac arrest survivor (cardiac arrest in previous three to 36-months)	Ability to engage in activities in English.	Stage 1.2 (focus groups): participants from 11 countries (represented by IAG membership)^b^	Advertised on patient/public-facing social media platforms
	Ability to participate in semi-structured interviews/focus groups using on-line technology or by telephone (option for individual interviews only)	Stage 1.3: (three-step interviews): participants from 11 countries (represented by IAG membership)^b^	Snowball sampling (supported by IAG membership)^b^
Stage 1.2.2 (focus groups)			
Health professionals with experience/expertise in the post-arrest care of cardiac arrest survivors: medical, nursing, physiotherapy, occupational therapy, psychology, cardiac and neurological rehabilitation		Stage 1.2.2 (focus groups): participants from 11 countries (represented by IAG membership)^b^	Convenience sample
			Advertised on healthcare professional-facing social media platforms
			Snowball sampling (supported by IAG membership)^b^

a Key supporter: defined as a family member/friend impacted by the cardiac arrest and/or subsequent recovery.

b International Advisory Group (‘PROM Buddies’) - an international group of clinicians, methodologists and public partners who are working collaboratively towards the development of the new PROM. The group includes survivors and key supporters (9), health professionals (20) and researchers (9) with expertise in post-cardiac arrest care and/or research, with representatives from eleven countries (Australia, Canada, Denmark, Finland, Germany, Italy, the Netherlands, Singapore, Sweden, United Kingdom, USA) (detailed in Appendix A).

Participants will be recruited from four countries (UK, Canada, USA, Australia) via advertisements placed on patient-facing social media platforms (Sudden Cardiac Arrest UK (SCA-UK), Canadian Resuscitation Outcomes Consortium (CanROC), Sudden Cardiac Arrest Survivors (SCAS) and the Sudden Cardiac Arrest Foundation (SCAF)(USA)), through members of the IAG, and snowball sampling.^28^, ^29^

All interviews will be conducted in English. Exclusion criteria include the inability to speak English or any co-morbidity that impairs the ability to participate in semi-structured interviews.

All interviews will be conducted remotely using online conference video digital technology or by telephone.

Informed by recent assessment guidance,^5^, ^8^ following the interview, all participants will be asked to complete a short generic measure of health status (the Short-Form 12-item Health Status Survey version 2 (SF-12v2® Health Survey|QualityMetric)^8^, ^30^ and a measure of mental wellbeing (Hospital Anxiety and Depression Scale (HADS)).^31^ Additionally, survivors will be interviewed to support completion of the modified Rankin Scale (mRS) as a measure of functional disability (mRS-9Q www.modifiedrankin.com)^8^, ^32^ and an abbreviated, telephone-administered version of the Montreal Cognitive Assessment (T-MoCA), as a measure of cognitive impairment (https://www.mocatest.org/).^33^ This data will allow us to describe the health status and degree of cognitive impairment in our participants, informing maximum variation of perspectives in the data.

#### Data collection

An interview schedule will be co-produced with our public partners to explore experiences of survival, recovery and survivorship, important outcomes and how these change over time, and concepts captured within the preliminary measurement framework. Their ability to recall and attribute certain aspects of health to the impact of cardiac arrest will also be explored.^9^ Interviews will be iterative with new insights explored in subsequent interviews. All interviews will be digitally audio-recorded and transcribed verbatim. Interview data will be analysed as interviews progress, informing the identification of potential themes, necessary modification of the interview schedule, and cessation of interviews once data saturation is achieved – i.e., no further aspects of health or potential domains are identified.^34^

#### Data analysis

We will draw on reflexive thematic analysis to support the development and classification of concepts of interest.^35^, ^36^ Supporting both inductive and deductive approaches towards theme development,^35^, ^36^ inductive analysis will support the discovery of emerging themes and categories from the data. Themes from the meta-ethnography and developing measurement framework will sensitize the researcher to the area and be used to prompt further exploration of themes, examine new areas of interest, and inform the analysis.^9^ The analytical process will involve data familiarisation and coding, whereby similar codes will be grouped together to identify categories. This will be followed by theme development, refinement, and naming. Data will be compared and contrasted throughout this process, and challenges within the data noted. NVivo software will be used to manage the data. Rigour will be demonstrated through immersion in the data, an audit trail of decisions made and description of the sample and context to aid transferability of the findings.^37^ The thematic framework from the interviews, meta- ethnography and HRQoL and recovery concepts from the literature will then be compared and contrasted.^11^ All concepts of interest will be arranged into overarching themes (‘domains’), each of which will contain multiple sub-themes (‘sub-domains’), informing further refinement of the developing measurement framework.^11^

#### Refining and confirming the measurement framework

Working collaboratively and iteratively, the core team, public partners and IAG will review the developing measurement framework and develop candidate questions (‘items’) that align with the proposed domains.^9^, ^10^, ^13^, ^19^ Reviews of existing measures of recovery, survivorship and HRQoL will be sought, item content of measures checked for relevance, and potential items identified and/or modified. Where necessary, and informed by the qualitative data, new items which capture the language of survivors will be crafted.

The comprehensibility and relevance of the developing measurement framework and candidate items will be further tested with multiple, external stakeholders – survivors, key supporters, and health professionals – participating in focus groups (stage 1.2).

### Confirming the measurement framework and refining items

#### Focus groups with survivors and key supporters

Using the networks and criteria described in stage 1.1 (Table 1), a convenience sample of survivors and their key supporters will be recruited from up to 11 countries (represented by IAG members), supporting the exploration of cultural differences in experiences. Semi-structured focus groups will be digitally hosted, moderated, and informed by good practice recommendations.^9^, ^38^ A focus group topic guide will be co-produced with our public partners. Participants will receive a copy of the developing measurement framework and candidate items in advance.

Focus group will have three components:

(1) First, findings from the literature reviews and qualitative interviews (stage 1) will be summarised, and the developing measurement framework presented. Participants will be invited to discuss the measurement framework in light of their own experiences of cardiac arrest recovery, highlighting where relevant outcomes may be missing;
(2) Participants will then rank the outcomes for importance, reaching agreement on those that should be included in the new measure; and
(3) Finally, participants will explore the relevance, resonance, acceptability and suitability of candidate items. This will also include questionnaire format and layout, item structure, recall period, and mode of completion (e.g., self, interview and proxy).

These tasks will be cognitively demanding, hence it is likely that participants will represent the more cognitively able. However, the involvement of key supporters will facilitate the participation of those who may be less able. Moreover, the involvement of healthcare professionals in subsequent focus groups/interviews will further mitigate the risk that the needs or experiences of survivors with lower degress of functional disability or cognitive impairment are insufficiently considered. Data will be analysed following each focus group, supporting an iterative process whereby ideas and concepts identified with earlier groups can be further explored.^9^

Each group will be audio-recorded, transcribed verbatim, and managed using NVivo software. Informed by the measurement framework and PROM design requirements, data will be thematically analysed,^19^, ^38^, ^39^ highlighting where there is both agreement and disagreement, and where further refinement is required.^9^, ^19^, ^38^

#### Focus groups/interviews with healthcare professionals

Accessing available networks, we will sample healthcare professionals involved in the post-arrest care of cardiac arrest survivors (Table 1). Focus groups (or individual interviews) will be conducted and hosted concurrently.

Modifications to the framework and candidate items will be confirmed between members of the core team, PPs, and IAG (Fig. 1). A questionnaire will then be developed, with candidate items grouped into conceptually similar domains. All item iterations will be tracked, with changes and justifications tabulated.^9^, ^10^

### Pre-testing the developing PROM and confirming content validity

Finally, the questionnaire will be further refined through an iterative process of semi-structured interviews with survivors and key supporters.^10^, ^19^, ^39^, ^40^

Multi-round, three-step test interviews (TSTI)^41^ will combine ‘thinking aloud’ and ‘verbal probing’ techniques to explore structure, comprehension, interpretation and completion problems, and to confirm comprehensiveness.^40^

All interviews will be conducted online and audio-digitally recorded, with observational notes made based on verbal and non-verbal cues. Concerns or problematic items will be recorded. Several interview rounds will be conducted, with up to ten participants per round. The analysis will consider if the participant: (i) understood the item; (ii) could retrieve the necessary information from memory; (iii) was able to make a judgment; and (iv) could select an appropriate response option.^39^, ^42^

Item modification will be iterative, informed by the results of each round, and collaboratively discussed between members of the core team, PPs, and IAG. Transparency in the decision-making process for item retention, modification or rejection will be supported by the application of an item assessment checklist.^9^, ^19^ Interviews will continue until no further issues are identified. The reading level of the questionnaire will be assessed after each round.^43^

Participants will be recruited from multiple countries (Table 1), contributing to the cross-cultural input. Data will be transcribed verbatim and managed using NVivo software.

At the end of this stage, the final list of candidate items for the new long-form CASHQoL will be confirmed.

#### Translation

Once the short-form version of the PROM is finalised (following stage 2.1 – Fig. 1), translations will be developed as informed by international guidelines^44^ and subjected to further testing.

#### Stage 2: Comprehensive psychometric and qualitative evaluation of the CASHQoL

Stage 2 will inform item refinement and reduction (Stage 2.1) and psychometric testing of the final measure (Stage 2.2).^10^, ^13^, ^14^, ^15^ Since funding and ethical approval is yet to be sought for this stage, an overview is provided.

In stage 2.1, cross-sectional completion of the CASHQoL by a large cohort of adult survivors will inform a preliminary psychometric evaluation.^14^, ^15^, ^45^ Qualitative feedback will also be sought.^22^ Data from the qualitative and psychometric (Rasch Measurement Theory (RMT)) analyses will inform the iterative refinement and removal of poorly functioning items; this will cease once the RMT statistics for retained items are within an acceptable range.^46^, ^47^, ^48^ The optimal number of retained items will be underpinned by the distribution of items across the concept of interest, and retention of the measure's content validity, producing a short-form version of the measure. Scoring guidance will be produced. To confirm that the measure’s content validity has been retained, additional semi-structured interviews will be conducted. All changes will be discussed and confirmed with the core team, PP, and IAG members.

Stage 2.2 will involve completion of the short-form CASHQoL by a further cohort of adult survivors on up to three occasions, supporting additional tests of measurement quality (data quality, interpretation, reliability, validity, and responsiveness) and acceptability.^14^, ^15^

Following this crucial stage in development, the refined CASHQoL will be ready for use within routine practice, cardiac arrest registries, and clinical research settings.

## Ethics

This study is co-ordinated at Warwick Medical School, Warwick University (Coventry, England) where it has ethical approval for stage one (Ethical approval: University of Warwick Biomedical & Scientific Research Ethics Committee (BSREC 22/20-21 granted 10/11/20)).

## Dissemination

The active collaboration with survivors, their key supporters, and an international advisory group, throughout the development process, will encourage innovation, reflection, and negotiation at key points of the development process. This will enhance the community ‘ownership’ and impact of the CASHQoL, supporting future use. The results from all key development stages will be shared through clinical and survivor organisations, networks, conferences, and publications.

## Discussion

The current reliance on generic measures to assess the perspective of cardiac arrest survivors is likely to underestimate the wide-ranging impact of survival.^1^, ^4^, ^11^ Survival, and the challenges of survivorship^1^, demand that we better understand an individual’s health and wellbeing following a critical illness. There are clear parallels to health-related quality of life – that is, understanding the perceived changes in emotional, physical and social well-being over time.^49^ The proposed PROM co-development process will help to define a measure that is specific to how survivors feel, function, and live their lives following cardiac arrest, and has resonance with the community; how this is defined, in terms of survivorship or health-related quality of life, will be a focus of stage 1. The co-development and uptake of the CASHQoL will ensure that survivor-important outcomes are included in research, registries and clinical practice, supporting the development of an informed evidence-base that values the experience of survivors.

A limitation of PROM development is that survivors with co-morbidities that impair their ability to engage in qualitative activities, e.g., due to severe cognitive impairment or fatigue, will not be included. However, their perspectives will be explored through key supporters. Moreover, the impact of such co-morbidities on PROM completion will be considered throughout the development process, influencing decisions regarding PROM length and delivery mode. The development process benefits from the collaborative engagement with key, international stakeholders – i.e., survivors, their key supporters, clinicians and methodologists –, affording a sense of both community and cultural validity to the PROM and a strong foundation for future translation activities. However, whilst the international literature will be integral to the development process, proposed participants will be reflective of high-income economies, which may limit the reach of the PROM.

A well-developed, co-produced PROM that is high quality, relevant and implementable across different settings will contribute essential, survivor-derived evidence to our understanding of recovery and long-term survivorship.

## Competing interests

KH, NP, CS, ET, HP, KD, PS, DE have no competing interests to declare.

KC is associate editor of Resuscitation Plus. GP is the Editor-in-Chief of Resuscitation Plus.

SA received significant funding from the National Institute of Health, National Heart, Lung, and Blood Institute R56/R01 HL153311.

## Funding statement

This work was supported by the Resuscitation Council UK (RCUK (Grant No ID: 2020-1462595057) and the Institute of Advanced Studies (IAS), Warwick University (IAS/23091/20).

## Authors’ contributions

KH is the Chief Investigator on this study and conceived the study with GDP. KH, CS, NP, ET, HP, PS, DE, KC, SA, and GP designed the project and lead the development of the protocol. PS, DE and other public partners contributed to the protocol development. All members of the IAG contributed to the protocol development. KH lead the writing of the manuscript. All authors and members of the IAG read and approved the final manuscript.

## Figures and Tables

**Table:** 

	**Clinician**	**Methodologist**	**Public Partners (PP)**
**Core Research Team**			
	Gavin Perkins (clinician)	Charly Southern (doctoral student)	Paul Swindell (survivor; Chair, Sudden Cardiac Arrest – UK (SCA-UK)
	Keith Couper (nurse)	Kirstie Haywood (PI)	David Jeffery (survivor)
	Sachin Agarwal (neurocritical care clinician) (USA)	Liz Tutton (qualitative)	Anne Brookes (survivor)
		Nathan Pearson (PROM development)	John Long (advocate)
		Helen Parsons (psychometrics)	Barry Williams (partner; advocate)
		Katie Dainty (qualitative) (Canada)	David Ellard (survivor; qualitative research)
N = 15	3	6	6
**International Advisory Group**			
***Europe***			
Denmark (1)	Vicki Joshi (physical therapy)		
England (4)	Tom Keeble (cardiologist)		
	Diane Playford (rehabilitation clinician)		
	Angela Hartley (cardiac rehabilitation nurse)		
	Marco Mion (clinical psychologist)		
Finland (1)	Maaret Castren (clinician)		
Germany (1)	Jan-Thorsten Graesner (clinician)		
Italy (1)	Federico Semeraro (clinician)		
Netherlands (2)	Veronique Moulaert (rehabilitation clinician)		
	Paulien Goossens (rehabilitation clinician)		
Sweden (3)	Gisela Lilja (occupational therapy)	Kristofer Arestedt (psychometrics)	
	Johnan Israelsson (nurse)		
***North America***			
Canada (2)	Laurie Morrison (emergency medicine)	Theresa Aves (trial manager; psychometrics)	Katrysha Gellis (survivor)
USA (4)	Kelly Sawyer (emergency medicine)		Rob Hoadley (survivor)
	Clifton Callaway (emergency medicine)		Jasmine Wylie (survivor; Sudden Cardiac Arrest Survivors (Face Book))
***Australasia***			
Australia (2)	Janet Bray (nurse)	Karen Smith (epidemiology; statistics)	
Singapore (1)	Marcus Ong Eng Hock (clinician)		
N = 23	17	3	3
***Total = 38***	***20***	***9***	***9***

a PROM Buddies: an international group of clinicians, methodologists and public partners who are working collaboratively towards the development of the new PROM.

**Table:** 

Name (alphabetical)	Affiliation	Country
Kristofer Arestedt	Faculty of Health and Life Sciences, Linnaeus University, Universitetsplatsen 1, SE-39231	Sweden
Theresa Aves	Division of Cardiology, St. Michael’s Hospital, 30 Bond Street, Toronto, Ontario, M5B 1W8	Canada
Janet Bray	Department of Epidemiology and Preventive Medicine, Monash University	Australia
Anne Brooks*	Warwick Medical School, University of Warwick, Gibbet Hill, Coventry, CV4 7AL	England
Clifton Callaway	University of Pittsburgh, School of Medicine, Pittsburgh, PA 15260	USA
Maaret Castren	Department of Emergency Medicine and Services, Helsinki and Helsinki University, Helsinki, Finland	Finland
Marcus Eng Hock Ong	Singapore General Hospital, Duke-NUS Medical School	Singapore
Katrysha Gellis*	Living Proof CPR Training, Toronto Ontario	Canada
Paulien H. Goossens	Merem medische revalidatie, Soestdijkerstraatweg 129, 1213VX Hilversum, The Netherlands	The Netherlands
Jan-Thorsten Graesner	Institute for Emergency Medicine, University Hospital Schleswig-Holstein, Kiel, Germany	Germany
Angela Hartley	Clinical Exercise, 35 Rivermead, East Molesey, Surrey KT8 9AZ, United Kingdom	England
Rob Hoadley*	Sudden Cardiac Arrest Foundation, 7500 Brooktree Road, Wexford, PA 15090. USA	USA
Johan Israelsson	Department of Internal Medicine, Kalmar County Hospital, Region Kalmar County, Kalmar, Sweden	Sweden
David Jeffrey*	Warwick Medical School, University of Warwick, Gibbet Hill, Coventry, CV4 7AL	England
Vicky Joshi	REHPA, The Danish Knowledge Centre for Rehabilitation and Palliative Care, University of Southern Denmark, Vestergade 17, 5800 Nyborg	Denmark
Thomas R. Keeble	1. Essex Cardiothoracic Centre, Basildon, Essex, UK 2. MTRC, Anglia Ruskin School of Medicine, Chelmsford, UK	England
Gisela Lilja	Neurology, Department of Clinical Sciences Lund, Lund University, Skane University Hospital, Lund, Sweden	Sweden
John Long*	Warwick Medical School, University of Warwick, Gibbet Hill, Coventry, CV4 7AL	England
Marco Mion	1. MTRC, Anglia Ruskin School of Medicine, Chelmsford, Essex, UK 2. Essex Cardiothoracic centre, MSE Trust, Basildon, Essex, SS16 5NL	England
Laurie J Morrison	Emergency Medicine Division of Emergency Medicine, Department of Medicine, University of Toronto Scientist, Emergency Services, Sunnybrook Health Sciences Centre, C753, 2075 Bayview Ave. Toronto ON M4N 3M5, Canada	Canada
Veronique R.M. Moulaert	University of Groningen, University Medical Center Groningen, Department of Rehabilitation Medicine, Groningen, The Netherlands. PO Box 30001, 9700 RB Groningen, The Netherlands	The Netherlands
Diane Playford	Division of Health Sciences, Warwick Medical School, , University of Warwick, Gibbet Hill, Coventry, CV4 7AL	England
Kelly Sawyer	University of Pittsburgh, School of Medicine, Pittsburgh, PA 15260	USA
Federico Semeraro	Department of Anaesthesia, Intensive Care and EMS, Ospedale Maggiore Carlo Alberto Pizzardi, 40133 Bologna, Italy	Italy
Karen Smith	1. Director Centre for Research and Evaluation, Ambulance Victoria, Australia 2. Adjunct Professor Department of Epidemiology and Preventive Medicine and Department of Paramedicine, Monash University, Melbourne Australia	Australia
Barry Williams*	Warwick Medical School, University of Warwick, Gibbet Hill, Coventry, CV4 7AL	England
Jasmine Wylie*	Sudden Cardiac Arrest Survivors (Face Book) https://www.facebook.com/groups/scasurvivors	USA
